# Die Bedeutung Früher Hilfen in Zeiten gesellschaftlicher Herausforderungen und Krisen

**DOI:** 10.1007/s00103-024-03980-9

**Published:** 2024-12-03

**Authors:** Ilona Renner, Ulrike Lux, Ursula von Rüden

**Affiliations:** 1 Nationales Zentrum Frühe Hilfen, Bundeszentrale für gesundheitliche Aufklärung, Maarweg 149–161, 50825 Köln, Deutschland; 2 Nationales Zentrum Frühe Hilfen, Deutsches Jugendinstitut e. V., Nockherstraße 2, 81541 München, Deutschland; 3 Abteilung Q – Forschung, Zielgruppen, Lebenslagen, Bundeszentrale für gesundheitliche Aufklärung, Maarweg 149–161, 50825 Köln, Deutschland

„… weil ich da so tief drin war in diesem Sumpf … Da war jemand da, der hat mir auch mal zugehört und nicht gleich irgendwie das so verurteilt meine Gedanken, oder meine Gefühle und so. Sondern, ja, war halt einfach da“ [1].

In der frühen Kindheit werden Weichen gestellt, die sich lebenslang auf die körperliche und seelische Gesundheit von Kindern auswirken können. Die Familie ist der Ort, an dem ein Kind, beginnend bereits in der Schwangerschaft, vielfältigen Reizen aus der sozialen und dinglichen Umwelt ausgesetzt ist, sie aktiv verarbeitet und Anpassungsstrategien entwickelt und erprobt. Diese frühesten Erfahrungen prägen teils lebenslang wirkende sozial- und gesundheitsrelevante Verhaltens- und Einstellungsmuster. Ist eine Familie psychosozial belastet, kann sich dies unmittelbar auf die Möglichkeiten eines Kindes auswirken, gesund aufzuwachsen. Deshalb unterstützen die Frühen Hilfen werdende Eltern und Familien von Anfang an.

Die Frühen Hilfen im deutschsprachigen Raum umfassen Beratung, Vermittlung und praktische Unterstützungsleistungen, die in kommunalen Netzwerken gebündelt sind. Zusammen mit Initiativen aus Ländern und Kommunen sowie von freien Trägern hat in Deutschland die Bundesstiftung Frühe Hilfen maßgeblich dazu beigetragen, dass die Frühen Hilfen heute flächendeckend ausgebaut sind [2]. Die Frühen Hilfen sind sektorenübergreifend angelegt. Sie verbinden insbesondere die verschiedenen Einrichtungen des Gesundheitswesens mit der Kinder- und Jugendhilfe, wobei Akteure weiterer Sektoren, wie bspw. die Schwangerschaftsberatungsstellen, in vielen Kommunen Teil der Netzwerke sind. Die interprofessionelle Kooperation soll Familien einen stigmatisierungsarmen Zugang zu psychosozialer Unterstützung ermöglichen – Unterstützung die passgenau auf ihre individuellen Bedarfe zugeschnitten ist. Damit tragen die Frühen Hilfen dazu bei, negative Auswirkungen familialer Belastungen, wie bspw. das Aufwachsen in Armut oder mit einer elterlichen psychischen Erkrankung, auf die kindliche Gesundheit und Entwicklung abzumildern.

Die letzten Jahre sind von vielfältigen Herausforderungen und Krisen geprägt. Die Corona-Pandemie wurde vielfach als schwierige Zeit erlebt – insbesondere von Familien, die ohnehin schon psychosozial belastet waren. Unmittelbar danach folgten der Krieg in der Ukraine, eine erneute größere Fluchtbewegung sowie die Energiekrise und weiterhin bestanden Sorgen im Zusammenhang mit dem Klimawandel. Dies wird von vielen Familien als dauerhafte, multiple Krisensituation wahrgenommen. Die Frühen Hilfen haben auf diese Herausforderungen flexibel reagiert. Trotz Kontaktbeschränkungen wurden Angebote während der Corona-Pandemie auf unterschiedlichste Weise aufrechterhalten [3, 4] und für Geflüchtete angepasst oder sogar erweitert [5]. Die (notwendigen) Weiterentwicklungen sind damit jedoch noch nicht am Ende. Die Frühen Hilfen bleiben ein lebendiges System mit familienunterstützenden Angeboten, die nah an den Bedarfen der Familien orientiert und in die kommunalen Versorgungsstrukturen eingebettet sind.

In Anbetracht der hier nur knapp skizzierten gesellschaftlichen Entwicklungen und Umbrüche sollen im vorliegenden Heft Kernthemen der Frühen Hilfen, Perspektiven und aktuelle Entwicklungen in den Blick genommen werden.

Im ersten Teil des Themenheftes werden die Situation und Bedarfslage von Familien mit kleinen Kindern aus Perspektive der sozial- und gesundheitswissenschaftlichen Empirie beleuchtet. Auf Basis von 5,14 Mio. Datensätzen von Frauen, die zwischen 2017 und 2022 bei der Krankenkasse BARMER versichert waren, beantworten *D. Hertle et al.* die provozierende Frage, ob Mutterwerden krank macht. Während in diesem Beitrag psychische Erkrankungen im Fokus stehen, geben *D. Chakraverty et al.*, basierend auf qualitativem Interviewdatenmaterial, einen Einblick in die Lebenswirklichkeit von Müttern, die aus der Ukraine nach Deutschland geflüchtet sind. Diese empirischen Studien zur aktuellen Situation der Familien werden von *J. Schoierer et al.* um Reflexionen dazu ergänzt, welche Bedeutung der Klimawandel aktuell und auch in näherer Zukunft für die Kinder- und Familiengesundheit hat bzw. haben wird und wie die Gesellschaft mit unterschiedlichen Anpassungsmaßnahmen darauf reagieren könnte.

Im zweiten Teil steht der Zusammenhang zwischen der elterlichen und der kindlichen psychischen Gesundheit im Mittelpunkt. *A‑L. Zietlow und L. Krumpholtz* geben einen Überblick über Mechanismen, die der transgenerationalen Übertragung psychischer Erkrankungen von Eltern auf Kinder zugrunde liegen können. *M. Hänelt et al.* berichten dazu empirische Ergebnisse einer aktuellen bundesweit repräsentativen Studie mit 7818 Säuglingen und Kleinkindern.

Im dritten Teil beschreiben *M. Weigl und S. Haas* die Entwicklung und den aktuellen Stand des Ausbaus der Frühen Hilfen im deutschsprachigen Raum. Sie identifizieren durchaus unterschiedliche Wege in den 5 untersuchten Ländern, um dasselbe Ziel zu erreichen. Die Frühen Hilfen sind nur dann wirksam, wenn sie schnell und flexibel auf gesellschaftliche Herausforderungen reagieren können. *U. Thyen *skizziert in einem weiteren Beitrag die Geschichte der Frühen Hilfen seit ihren Ursprüngen, sie beleuchtet zentrale Wissensbestände und zeigt aktuelle Entwicklungsperspektiven auf.

Abschließend geht es im vierten Teil um empirische Evidenz für die Wirkung von Komponenten Früher Hilfen. *S. Weyers und S. Götz* prüfen, inwieweit Schuleingangsuntersuchungen eine Möglichkeit bieten, die Wirkung früher Präventions- und Vermittlungsangebote zu untersuchen. Sie skizzieren Chancen und Herausforderungen von Wirkungsanalysen. Dass eine anspruchsvolle Wirkungsstudie auch im Feld der Frühen Hilfen umgesetzt werden kann, zeigen *C. Schlett et al. *In einem quasiexperimentellen Design untersuchen sie, ob sektorenübergreifende Kooperation die Identifikation familialer Belastungen in der kinderärztlichen Praxis verbessern kann.

Das vorliegende Themenheft gibt einen Einblick in den Stand der Frühen Hilfen, die aktuellen Themen, Perspektiven und Entwicklungslinien. Neben den Herausforderungen für Familien, teils als Reaktion auf multiple Krisen, werden auch die Entwicklungspotenziale Früher Hilfen in den Blick genommen. Dies ist zentral, damit psychosozial belastete Familien auch künftig frühzeitig unterstützt werden können und die Frühen Hilfen weiterhin zu einem gesunden und gewaltfreien Aufwachsen von Kindern beitragen können.
